# Clever-1 positive macrophages in breast cancer

**DOI:** 10.1007/s10549-022-06683-4

**Published:** 2022-08-02

**Authors:** Minna Mutka, Reetta Virtakoivu, Kristiina Joensuu, Maija Hollmén, Päivi Heikkilä

**Affiliations:** 1 Department of Pathology, HUSLAB, Helsinki University Hospital and University of Helsinki, N00290 Helsinki, Finland; 2 MediCity Research Laboratory, University of Turku, Turku, Finland; 3 University of Helsinki, N00290 Helsinki, Finland

**Keywords:** Clever-1, Breast cancer, Tumor-associated macrophages, Tumor infiltrating lymphocytes, Tumor stroma

## Abstract

**Purpose:**

Common Lymphatic Endothelial and Vascular Endothelial Receptor 1 (Clever-1) is expressed by a subset of immunosuppressive macrophages and targeting the receptor with therapeutic antibodies has been shown to activate T-cell-mediated anti-cancer immunity. The aim of this research was to study Clever-1 expression in breast cancer. Specifically, how Clever-1 + macrophages correlate with clinicopathologic factors, Tumor Infiltrating Lymphocytes (TILs) and prognosis.

**Methods:**

Tissue microarray blocks were made from 373 primary breast cancer operation specimens. Hematoxylin and Eosin (H&E-staining) and immunohistochemical staining with Clever-1, CD3, CD4 and CD8 antibodies were performed. Differences in quantities of Clever-1 + macrophages and TILs were analyzed. Clever-1 + cell numbers were correlated with 25-year follow-up survival data and with breast cancer clinicopathologic parameters.

**Results:**

Low numbers of intratumoral Clever-1 + cells were found to be an independent adverse prognostic sign. Increased numbers of Clever-1 + cells were found in high grade tumors and hormone receptor negative tumors. Tumors that had higher amounts of Clever-1 + cells also tended to have higher amounts of TILs.

**Conclusion:**

The association of intratumoral Clever-1 + macrophages with better prognosis might stem from the function of Clever as a scavenger receptor that modulates tumor stroma. The association of Clever-1 + macrophages with high number of TILs and better prognosis indicates that immunosuppression by M2 macrophages is not necessarily dampening adaptive immune responses but instead keeping them in control to avoid excess inflammation.

## Introduction

Common lymphatic endothelial and vascular endothelial receptor-1 (Clever-1, also called Stabilin-1 and Feel-1) is a type I transmembrane protein commonly expressed on non-continuous endothelium [1, 2] and a subset of alternatively activated M2 macrophages [3, 4]. In macrophages, Clever-1 is a scavenger receptor [4, 5] that participates in the scavenging of pathogens, apoptotic cells and molecules [6]. In the endothelium it takes part in lymphocyte, granulocyte and monocyte trafficking and transmigration and can participate in cancer cell migration in the lymphatics [7]. It can also have functions that modulate the immune response [8, 9] and angiogenesis [6]. The gene encoding Clever-1 is called *STAB1* [10].

Macrophages are the most abundant benign cells in tumors [11]. Clever-1 is expressed on a subpopulation of alternatively activated macrophages that are immunosuppressive [12]. M2 macrophages are thought to be protumorigenic and essential for tumor progression. They enhance tumor invasiveness, angiogenesis and intravasation into the circulation [11, 13] and stimulate tumor cell proliferation [14]. They are anti-inflammatory and function by negatively affecting cytotoxic CD8 + T-cells [15, 16], and by promoting immunosuppressive regulatory T lymphocytes [17]. They inhibit proinflammatory Th1 type responses and promote Th2 type responses [12]. Clever-1 may participate in many of these functions directly, [12, 13, 15, 16] and therefore anti-Clever therapies are under development [18].

Clever-1 + macrophages have been recorded in solid cancers such as melanoma [19] and glioblastoma [20]. They associate with a worse prognosis in bladder carcinoma [21–23] and predict metastasis and risk of recurrence in oral cavity squamous cell carcinoma [24]. Clever-1 + macrophages in gastric cancer predict worse prognosis in tumors with a high T-lymphocyte count [25] or in early stage tumors [26]. High numbers of peritumoral Clever-1 + macrophages in colorectal carcinomas correlate with a better prognosis in early stages, but in the metastatic stage, high intratumoral counts correlate with a worse prognosis [27]. Similarly, one study on bladder cancer has shown that only intratumoral, but not stromal, Clever-1 + macrophages correlate with a worse prognosis [28]. Thus, the evidence on the effect of Clever-1 + macrophages to prognosis is conflicting.

Very little is known about how Clever-1 + macrophages affect breast cancer prognosis and how they associate with different types of breast cancer. Clever-1 + macrophages were reported in 82% of breast carcinomas [29]. The removal of Clever-1 + macrophages inhibit growth of mammary adenocarcinoma in a mouse model [30]. One study showed no correlation between Clever-1 + macrophages and lymph node metastasis [31]. However, another study recorded a correlation between the amount of Clever-1 + macrophages in areas of dense fibrotic stroma in neoadjuvant treated breast cancer and the number of metastatic lymph nodes [32].

We have immunohistochemically investigated 373 cases of primary breast carcinoma for Clever-1 + macrophages. The first aim of this study was to correlate Clever-1 + macrophage numbers with the clinicopathologic features of breast cancer. The second aim was to investigate the relationship of Clever-1 + macrophages with tumor infiltrating lymphocytes (TILs). The third aim was to investigate how Clever-1 + macrophages affect prognosis.

## Methods

### Patients and tissue samples

The material consisted of all operated primary breast carcinomas in Helsinki University Hospital for the years 1991–1992. All cases were reviewed for tissue sufficiency and after removal of scarce samples, 373 cases remained, for information about the cases see Table 1.

**Table 1:** Clinicopathologic information of the cases

	No of cases	% cases
ER status		
Positive	314	85.8
Negative	52	14.2
PR status		
Positive	285	77.2
Negative	84	22.8
HER2 status		
Positive	48	13.4
Negative	309	86.6
Type of tumor		
ER + HER2-	253	72.1
ER + HER2 +	19	5.4
ER-HER2 +	28	8.0
TNBC	51	14.5
Mib-1 status		
Low	305	82.9
High	63	17.1
Histological type		
Ductal	279	74.8
Lobular	60	16.1
Other	34	9.1
Grade		
1	107	28.7
2	184	49.3
3	82	22.0
Size of tumor		
1–20 mm	225	60.3
≥ 20 mm	148	39.7
Lymph node status		
Positive	132	32.8
Negative	241	64.6
Stage		
0	6	1.6
1	232	62.2
2	125	33.5
3	9	2.2
4	1	0.3

*ER* estrogen receptor, *PR* progesterone receptor, *HER2* human epidermal growth factor receptor 2, *TNBC* triple negative breast cancer

After the selection of cases Hematoxylin and Eosin (H&E)-stained sections were prepared from the most representative blocks and scanned into digital images with Pannoramic Scan 150, Pannoramic Scan II or Pannoramic 250 Flash III (3DHISTECH, Budapest, Hungary). Images were digitally annotated with CaseViewer (3DHISTECH, Budapest, Hungary). Two 1 mm punches were annotated from the center and two from the periphery of each tumor. If the material was scarce, fewer annotations were made. The images were imported and subjected to software analysis, then overlayed with the donor block images, subsequently the layout of the Tissue Microarray (TMA) blocks was designed. TMA Grand Master (3DHISTECH, Budapest, Hungary) tissue microarrayer transferred the cores from the donor block to the recipient TMA blocks, where they were heat-sealed.

### Immunohistochemistry

Formalin-fixed paraffin-embedded tissue blocks were cut into 4-µm-thick sections. After deparaffinization, the slides were pretreated in a PT module (LabVision UK Ltd., Suffolk, UK) in Tris–EDTA pH 9.0 (100 °C for 24 min) and cooled to room temperature. Immunohistochemical stainings were done using the following antibodies: CD4 (dilution 1:500, clone 4B12, M7310, Dako), CD8 (dilution 1:100, clone C8/144B, M7103, Dako), ER-alfa (dilution 1:200, clone 6F11, MA5-13,304, Thermo fisher), PR-alfa (dilution 1:200*,* clone 636, M3569, Dako) and Ki67-1 (dilution 1:75, clone MIB1, M7240 DAKO). The polymer detection kit EnVision (K5007, Dako) was used in a LabVision Autostainer (Thermo Scientific, Fremont, CA). For Clever-1-staining proteinase K antigen retrieval and 2–7 AB (rat IgG) hybridoma medium (in-house, undiluted) was used as previously described [1, 6].

Slides for CD3 and Her2 were stained in Ventana Benchmark Ultra (Ventana/Roche, Tucson, AZ) using the following antibodies: CD3 (RTU, clone 2GV6, 790–4341, Ventana/Roche), HER2 (dilution 1:400, clone CB11, Novocastra). Pretreatment was performed with Ventana Cell Conditioning Solution CC1 (Roche, Tucson, AZ) at 98 °C for 64 min. The primary antibodies were incubated at 36 °C for 32–48 min (Her2 for 48 min, and CD3 for 40 min). OptiView DAB IHC Detection Kit (760–700 Ventana/Roche) was used for detection. The slides were counterstained with Mayer's hematoxylin and mounted in a mounting medium.

All tumors were tested for HER2 gene amplification using Inform HER2 Dual ISH in situ hybridization with Ventana Bechmark Ultra (Ventana/Roche, Tuscon, AZ). After triple pretreatment with solutions CC1 16 at 98 °C for 16 min (950–224, Ventana/Roche) + CC2 at 98 °C for 24 min (950-223, Ventana/Roche) and protease-3 at 37 °C for 16 min (780–4149, Ventana/Roche), the HER2 gene was targeted using a dinitrophenyl labeled probe and the chromosome 17 centromere was localized with a digoxigenin labeled probe (INFORM HER2 Dual ISH DNA Probe Cocktail, 780–4422, Roche/Ventana/Tuscon, AZ, USA 780-4422). HER2 was visualized as black signals with VENTANA ultraView Silver ISH DNP (SISH) Detection (760–098, Roche/Ventana/Tuscon, AZ, USA) and Chr17 as red signals with VENTANA ultraView Red ISH DIG Detection (780–4422, Roche/Ventana/Tuscon, AZ, USA).

**Fig. 1:**
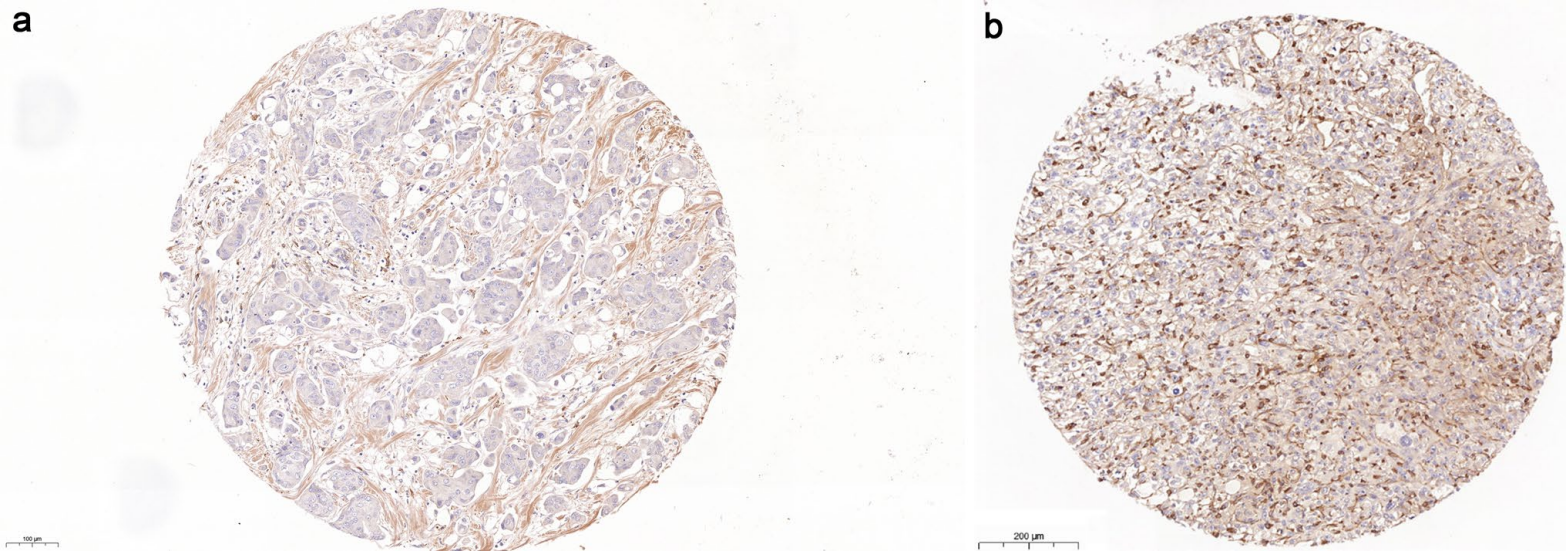
Clever-1 stainings. **a** Punch that was counted to have 37 Clever + positive macrophages. **b** Punch that was counted to have 490 Clever + positive macrophages

Clever-1 + macrophages were counted using the CaseViewer (3DHISTECH, Budapest, Hungary). Intratumoral and peritumoral Clever-1 + macrophages were counted separately by two persons blinded to each other's counts and from these separate counts mean numbers were calculated (Fig. 1).

CD3 + , CD4 + and CD8 + cells were evaluated according to and modifying the principles presented by the International TILs working group [33] and the International Immuno-Oncology Biomarkers Working Group [34]. Stromal scores were given by the above- mentioned working groups principles for H&E-sections and intratumoral and peritumoral scores were given by modifying these principles for each compartment. Intratumoral was defined as lymphocytes in direct contact with tumor cells, in contrast to stromal lymphocytes that reside in the tumor stroma without direct contact to tumor cells. Intratumoral (i) CD3 + and CD8 + percentages were defined as low if 0–3%, moderate if 4–9% and high if over 10%. Stromal (s) and peritumoral (p) CD3 + and CD8 + percentages were defined as low if 0–10%, moderate if 11–49% and high if 50–100%. As the CD4 + percentages were generally low, they were categorized into only two categories, low was defined as 0% for iCD4 + and 0–3% for sCD4 + and pCD4 + , high as 1–100% for iCD4 + and 4 –100% for sCD4 + and pCD4 + .

ER and PR were defined as negative when < 1% of tumor cell nuclei were positive and positive when ≥ 1% of tumor cell nuclei were positive. Ki-67 was categorized as being low when ≤ 20% of tumor cell nuclei in hot spots were positive, and high when > 20% were positive. Cases with positivity in HER2 in situ staining were categorized as positive for HER2.

### Statistics

Clever-1 + macrophage quantities were correlated with dichotomic clinicopathologic factors and CD4 + cell percentages using the Mann–Whitney-U-Test and with other clinicopathologic factors and CD3 + and CD8 + cell percentages using the Kruskal–Wallis Test. Cumulative survival was calculated using the Kaplan–Meier survivorship method and was analyzed by the log-rank test. A multivariate Cox proportional hazards model was used to estimate the adjusted hazard ratios with 95% confidence intervals (CIs), and to identify independent prognostic factors. *P*-values < 0.05 were considered significant.

## Results

### Clever-1 + macrophages according to breast cancer clinicopathologic factors

Means of the numbers of Clever-1 + cells in tumors according to different clinicopathological factors and the *p*-values for the significance of their differences are shown in Table 2.

**Table 2:** Mean numbers of Clever-1 + macrophages according to breast cancer clinicopathologic factors

Factor	Intratumoral clever + cells mean numbers	*p*-value	Peritumoral clever + cells mean numbers	*p*-value
ER status				
Positive	26.19	0.050*	40.13	0.004*
Negative	34.62		69.10	
PR status				
Positive	26.97	0.814	41.41	0.112
Negative	29.61		53.86	
Ki-67 status				
Low	25.10	0.026*	40.01	0.002*
High	38.85		64.15	
HER2 status				
Positive	47.19	0.993	34.14	0.273
Negative	43.65		30.84	
Tumor type				
ER + HER2-	24.15	0.281	37.25	0.001*
ER + HER2 +	31.58		43.13	
ER-HeR2 +	30.41		51.55	
TNBC	41.13		74.35	
Tumor histology				
Ductal carcinoma	28.21	0.314	45.36	0.001*
Lobular carcinoma	23.88		30.11	
Other	31.65		59.34	
Tumor grade				
G1	24.78	0.158	38.00	0.000*
G2	24.58		37.82	
G3	39.16		66.75	
Tumor size				
1–20 mm	24.81	0.028*	40.66	0.026*
> 20 mm	32.42		49.67	
Lymph node status				
Positive	41.68	0.873	26.00	0.931
Negative	45.68		28.86	
Stage				
Stage 0	36.58	0.335	67.45	0.070
Stage 1	28.83		45.41	
Stage 2	25.32		42.71	
Stage 3	35.38		25.22	
Stage 4	3.00		12.25	

Significant differences are marked with *. *ER* estrogen receptor, *PR* progesterone receptor, *HER2* human epidermal growth factor receptor 2, *TNBC* triple negative breast cancer

ER- tumors had more Clever-1 + macrophages both intra- and peritumorally. There were no significant differences between PR- and PR + tumors or HER2- and HER2 + tumors.

TN tumors had more Clever-1 + macrophages peritumorally than ER + HER- tumors (*p* = 0.001). No significant differences were seen in pairwise comparisons between the other tumor types, nor regarding the amounts of Clever-1 + macrophages intratumorally.

The Ki-67 high tumors had more Clever-1 + macrophages intra- and peritumorally than the Ki-67 low tumors.

Ductal (*p* = 0.001) and other types (*p* = 0.002) of carcinomas had significantly more peritumoral Clever-1 + macrophages than lobular carcinomas, but the difference between ductal and other types of carcinomas was not significant. Grade (G) 3 tumors had significantly more peritumoral Clever-1 + cells than G1 (*p* < 0.001) and G2 tumors (*p* = 0.001). There were no differences in the amounts of intratumoral Clever-1 + macrophages.

Tumors > 20 mm had more intra- and peritumoral Clever-1 + macrophages than smaller tumors.

Age of the patient, cancer stage or lymph node status at diagnosis did not correlate with Clever-1 + amounts.

### Clever-1 + macrophages correlate with CD3 + , CD8 + and CD4 + lymphocytes

Mean numbers of Clever-1 + macrophages according to CD3 + , CD8 + and CD4 + percentages and *p*-values for the significance of the differences can be seen in Table 3.

**Table 3:** Amount of Clever + macrophages according to Tils values

Tils	Clever center mean	*p*-value	Clever periphery mean	*p*-value
iCD3				
Low	26.43	0.002*	42.72	0.018*
Moderate	46.94		79.39	
High	48.65		65.5	
sCD3				
Low	24.55	< 0.001*	39.81	< 0.001*
Moderate	38.63		59.53	
High	60.47		94.42	
pCD3				
Low	24.31	0.050	38.61	< 0.001*
Moderate	34.41		52.82	
High	30.72		71.99	
iCD8				
Low	26.90	0.045*	42.70	0.004*
Moderate	38.06		78.86	
High	47.66		76.72	
sCD8				
Low	25.30	0.001*	41.03	0.001*
Moderate	45.22		68.92	
High	56.50		87.58	
pCD8				
Low	26.60	0.272	39.70	< 0.001*
Moderate	30.09		62.55	
High	30.92		64.47	
iCD4				
Low	24.33	0.001*	41.21	0.058
High	37.52		54.50	
sCD4				
Low	24.54	< 0.001*	39.93	< 0.001*
High	47.01		74.04	
pCD4				
Low	25.55	0.059	39.90	< 0.001*
High	32.33		60.12	

*I* intratumoral, *p* peritumoral/periphery, *s* stromal

Significant changes are marked with *

Tumors that had high percentages of iCD3 + cells had higher amounts of intratumoral Clever-1 + macrophages. In the pairwise comparisons, the difference was significant between those tumors with a low percentage of iCD3 + cells and those with a moderate percentage (*p* = 0.023). Peritumorally the overall differences were significant, but in pairwise comparisons no significant differences were recorded.

Higher iCD8 + percentages also meant higher Clever-1 + cell numbers. This was seen for overall intratumoral Clever-1 + cells, although this difference was not seen in pairwise comparisons. Tumors with moderate amounts of iCD8 + cells had significantly more Clever-1 + cells than tumors with low iCD8 + percentages (*p* = 0.017).

Tumors that had high percentages of sCD3 + cells also had higher numbers of both intratumoral and peritumoral Clever-1 + macrophages. Moreover, the difference was significant in comparisons with both tumors with a moderate percentage compared to low percentage (*p* = 0.009 for intratumoral and *p* = 0.002 for peritumoral) and high percentage compared to low (*p* = 0.001 for intratumoral and *p* = 0.001 for peritumoral). The same relationship was seen between sCD8 + cells and Clever-1 + macrophages, this difference was significant in pairwise comparisons only between tumors with a low and moderate percentage of sCD8 + cells (*p* = 0.003 for intratumoral and *p* = 0.009 for peritumoral Clever-1 + macrophages).

Higher numbers of Clever-1 + macrophages were seen peritumorally in tumors with higher percentages of pCD3 + and pCD8 + . This difference was significant between tumors with low and moderate percentages of pCD3 + cells (*p* < 0.001) and pCD8 + cells (*p* < 0.001) and low and high percentages of pCD3 + cells (*p* = 0.001). There were no significant differences in the numbers of Clever-1 + macrophages intratumorally.

The Clever-1 + macrophage amounts were higher in tumors with higher amounts of CD4 + cells. This was seen in all compartments for CD4 + cells and for intratumoral and peritumoral Clever-1 + macrophages.

### Abundance of intratumoral Clever-1 + macrophages are a favourable prognostic sign

**Fig. 2:**
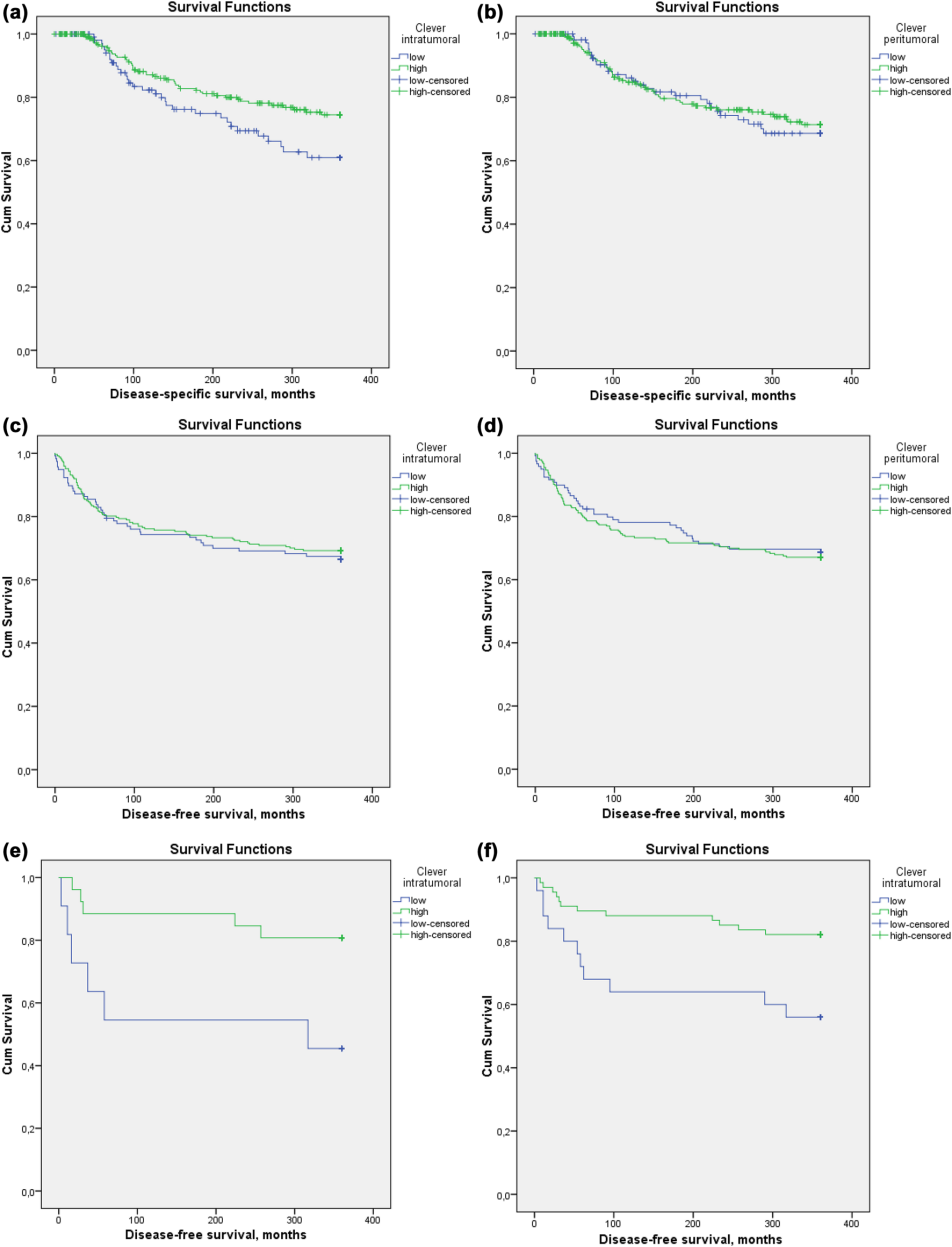
Kaplan–Meier curves that show patient disease-specific survival (DSS) and disease-free survival (DFS) in comparison to intratumoral and peritumoral Clever-1 + amounts. Figure created with SPSS 22.0 for Windows. **a** DSS in months according to intratumoral Clever-1 + amount *p* = 0.036. **b** DSS in months according to peritumoral Clever-1 + amount *p* = 0.749. **c** DFS in months according to intratumoral Clever-1 + amount *p* = 0.600. **d** DFS in months according to peritumoral Clever-1 + amount *p* = 0.709. **e** DFS in months according to intratumoral Clever-1 + amount in patients with high percentages of pCD3 *p* = 0.020. **f** DFS in months according to intratumoral Clever-1 + amount in patients with high percentages of pCD4 *p* = 0.008

Kaplan–Meier curves that show patient disease-specific survival (DSS) and disease-free survival (DFS) in comparison to intratumoral and peritumoral Clever-1 + amounts are seen in Fig. 2.

Patients with low amounts of intratumoral Clever-1 + macrophages had a shorter DSS than patients with higher amounts. No differences were seen in DFS in the entire population, but when analyzed separately patients with high amounts of pCD3 + cells or pCD4 + cells along with low amounts of Clever-1 + cells had shorter DFS. Peritumoral Clever-1 + macrophage counts did not affect survival.

For survival analysis, see Table 4. Univariate Cox proportional hazard analysis was done for Clever-1 + cells, TILs and clinicopathologic factors, and in these analyses intratumoral Clever-1 + cells, sCD8 + cells, sCD4 + cells, stage, size of tumor and lymph node status at diagnosis emerged as prognostic factors for DSS. These factors were chosen for multivariate Cox proportional hazard analysis. In this analysis large tumor size and low amounts of sCD8 + cells and intratumoral Clever-1 + cells were independent adverse prognostic signs.

**Table 4:** Survival analysis according to different breast tumor factors

Variable	Univariate				Multivariate					
	Disease-free survival		Disease-specific survival		Disease-free survival			Disease-spesific survival		
	Cox *p*-value	Hazard ratio	Cox *p*-value	Hazard ratio	Cox *p*-value	Hazard ratio	95% CI	Cox *p*-value	Hazard ratio	95% CI
Intratumoral Clever + cells low vs. high	0.601	0.902	**0.038**	**0.621**				**0.023**	**0.584**	**0.368–0.929**
Peritumoral Clever + cells low vs. high	0.709	1.077	0.749	0.927						
iCD3 + cells low vs. moderate or high	0.112	0.394	0.101	0.192						
sCD3 + cells low vs. moderate or high	0.149	0.654	0.062	0.478						
pCD3 + cells low vs. moderate or high	0.927	1.019	0.569	0.867						
iCD8 + cells low vs. moderate or high	0.268	0.523	0.142	0.228						
sCD8 + cells low vs. moderate or high	**0.019**	**0.343**	**0.019**	**0.185**	0.145	0.429	0.138–1.338	**0.027**	**0.179**	**0.039–0.824**
pCD8 + cells low vs. moderate or high	**0.016**	**0.551**	0.224	0.714	**0.021**	**0.538**	**0.318–0.910**			
iCD4 + cells low vs. high	0.385	1.199	0.929	1.023						
sCD4 + cells low vs. high	**0.017**	**0.394**	**0.044**	**0.395**	0.063	0.421	0.169–1.048	0.738	0.845	0.314–2.271
pCD4 + cells low vs. high	0.088	0.671	0.549	0.853						
ER + vs. ER-	0.636	1.132	0.719	1.136						
PR + vs PR-	**0.037**	**1.534**	0.603	1.157	**0.001**	**2.119**	**1.345–3.339**			
Ki-67 low vs. high	0.362	1.240	0.545	1.217						
HER2 + vs. -	0.205	0.721	0.298	0.702						
Type of tumor ER + HER2- vs any other	0.068	1.450	0.467	1.219						
Histology ductal vs. lobular or other	0.284	0.789	0.552	1.156						
Grade 1 vs. 2 or 3	**0.031**	**1.615**	0.154	1.435	0.067	1.545	0.971–2.460			
Size 0–20 vs. > 20	**0.001**	**1.794**	**0.005**	**1.880**	**0.032**	**1.546**	**1.038–2.302**	**0.004**	**1.987**	**1.243–3.176**
Stage 0 and 1 vs. 2, 3 or 4	**0.002**	**1.780**	**0.000**	**2.851**	0.400	0.634	0.219–1.835	0.366	1.637	0.562–4.764
Lymph node status negative vs. positive	**0.002**	**1.793**	**0.000**	**2.754**	0.094	2.497	0.857–7.281	0.405	1.573	0.541–4.569
Age of patient 0–50 vs. > 50	**0.043**	**0.689**	0.916	1.024	**0.014**	**0.611**	**0.412–0.905**			

*I* intratumoral, *s* stromal, *p* peripheral, *ER* estrogen receptor, *PR* progesterone receptor, *HER2* human epidermal growth factor receptor 2

Significant values in bold

In a similar manner, univariate analysis was performed for DFS. In this analysis Clever-1 + macrophages were not found to be of prognostic significance. Tumor size, stage, lymph node status and patient age at diagnosis, grade, PR status, sCD8 + cells, pCD8 + cells and sCD4 + cells were prognostic factors and chosen for multivariate analysis. In multivariate analysis, independent factors for shorter DFS were size over 20 mm, younger age, PR negativity and low amounts of pCD8 + cells.

## Discussion

We studied Clever-1 + macrophages in 373 breast cancer specimens. To our knowledge this is the first study to investigate Clever-1 + macrophages in a large cohort of breast cancer cases with a long follow-up time. It therefore provides many interesting insights into the distribution and significance of Clever-1 + macrophages and in breast cancer.

We showed that intratumoral Clever-1 + macrophages correlated with a better DSS, whereas peritumoral Clever-1 + macrophages did not affect prognosis. Although Clever-1 + macrophages generally have been associated with a worse prognosis [21–24, 26], peritumoral Clever-1 + macrophages have been associated with a better prognosis in colorectal carcinoma in early stage [27]. The location of Clever-1 + macrophages seem to be important as intratumoral, unlike stromal, Clever-1 + macrophages have been associated with a higher grade and stage and worse prognosis in bladder cancer [28].

One explanation for why intratumoral Clever-1 + macrophages are associated with a better prognosis, may be related to the function of Clever-1 as a scavenger receptor. Acting locally by scavenging some extracellular components and secreting others, Clever-1 modulates the tumor stroma and its growth milieu.

Clever-1 mediates the endocytosis and lysosomal degradation of Secreted Protein Acidic and Rich in Cysteine (SPARC). SPARC is a soluble extracellular matrix protein that participates in tissue remodeling and binds growth factors thereby inhibiting their function [3, 4, 35]. By affecting the extracellular matrix composition SPARC can potentially affect cancer growth [20]. Research has shown that greater amounts of SPARC in breast cancer in mice inhibit cancer growth [30, 36]. On the other hand, SPARC downregulation in malignant gliomas inhibits cancer cell migration and invasiveness [20] and in lung cancer SPARC has been associated with increased growth and metastatic potential [36]. The exact mechanisms and effect of SPARC is therefore unknown. Clever-1 also participates in the recycling and secretion of chitinase-like proteins that have properties of both cytokines and growth factors. YKL-39 is one of these and is associated with a worse prognosis [37], whereas another, SI-CLP, seems to inhibit growth of mammary adenocarcinoma in mouse models [38]. Yet other target substances of Clever-1 exist and can participate in Clever-modulated ECM remodeling affecting the cancer microenvironment.

On the other hand, Clever-1 is a marker of M2 alternatively activated macrophages. These macrophages are thought to be protumorigenic. Higher amounts of CD68 + cells in breast cancer have been associated with a worse prognosis and M2 macrophages are known to stimulate cancer proliferation, angiogenesis and immunosuppression. The effects of macrophages on prognosis have, however, been contradictory [39]. A lack of Clever-1 has been associated with slower cancer growth in mouse models [17]. This seems contradictory to the association of Clever-1 + macrophages in this study with a better prognosis. It is still worth remembering that only a third of type M2 Tumor Associated Macrophages (TAMs) in cancer are Clever-1 + and Clever is a late marker of M2 macrophage polarization [13, 17].

There are many known effects of Clever-1 + cells and M2 macrophages on the cancer immune reaction. Type − 2 macrophages induce immunosuppressive regulatory T-cells (Tregs) by direct contact and with cytokines [17] and they promote an immunosuppressive Th2 type anti-inflammatory reaction [12]. Anti-Clever-1-therapies have enhanced antitumor CD8 + reactions [15, 18]. According to some studies, Clever-1 directly participates in this process, instead of just being a marker of M2 polarization [12, 15]. Clever-1 + macrophages might bind to CD8 + and CD20 + lymphocytes and thereby inhibit their function. In TNBC with *STAB1* overexpression, there seems to be a dysfunction of CD8 + lymphocytes and in some cancers, which are *STAB1* high, CD8 + lymphocytes have correlated with a worse prognosis, whereas in cancers with low *STAB1*, they have correlated with a good prognosis. Additionally Clever-1 + mediated lysosomal degradation of products affects antigen presentation [16]. It is also possible that Clever-1 + macrophages mediate lymphocyte extravasation and accumulation, which enhances tumor cell killing. In this study, the amounts of TILs positively correlated with Clever-1 + macrophages. As intratumoral Clever-1 + macrophages correlated with a better prognosis, it would seem, that they were not able to entirely inhibit the function of cytotoxic T-cells.

CD8 + lymphocytes are cytotoxic cells that engage in tumor killing by inducing cytolysis [40], which explains their positive effect on prognosis, a finding well in line with previous studies [41–45]. PR negativity associates with a worse prognosis in ER + breast cancer, and with resistance to endocrine treatment [46, 47].

In this study, higher amounts of Clever-1 + macrophages correlated with a higher grade, ER negativity and larger size, but not with HER2 status. This finding is well in line with previous studies concerning TAMs in breast cancer [39]. The same tumors also had higher amounts of TILs, and in studies on TILs, they have been associated with many of the same factors as TAMs, although HER2 + tumors tend to have higher amounts of TILs [48, 49]. TILs are associated with a better prognosis in at least TNBC and HER2 + breast cancer [50]. According to our results the same tumors that attract TILs also attract Clever-1 + macrophages, creating an immune-rich phenotype. Clever-1 + macrophages can attract TILs, but they can also modulate their function.

This study has all the limitations known for a retrospective study. TAMs tend to cluster, which limits the accuracy of TMAs in this context [21]. This limitation has been considered, and therefore several sample punches per tumor were taken for study.

## Data Availability

The datasets generated during and/or analysed during the current study are available from the corresponding author on reasonable request.
